# The role of the incretin GIP in inflammation

**DOI:** 10.1007/s40618-025-02719-w

**Published:** 2025-10-13

**Authors:** Giada Rossi, Loredana Bucciarelli, Vincenzo Cimino, Paolo Fiorina

**Affiliations:** 1 International Center for T1D, Pediatric Clinical Research Center Romeo ed Enrica Invernizzi, DIBIC, Università di Milano, Milan, Italy; 2 Division of Endocrinology, ASST Fatebenefratelli Sacco, Milan, Italy; 3 Pio Albergo Trivulzio, Milan, Italy; 4 Nephrology Division, Boston Children’s Hospital, Harvard Medical School, 300 Longwood Ave. Enders Building, Boston, 02115 MA USA; 5 Transplantation Research Center, Brigham and Women’s Hospital, Harvard Medical School, Boston, MA USA

**Keywords:** GIP, Inflammation, GIP agonists, GIP antagonists, Diabetes mellitus, Obesity

## Abstract

Glucose-dependent insulinotropic polypeptide (GIP) is an incretin hormone traditionally known for its insulinotropic and adipogenic effects. However, its role in immune modulation and inflammation has recently gained attention, particularly in the context of metabolic diseases. By conducting a comprehensive search into the scientific literature since the discovery of GIP hormone, this review examines the biological evidences linking GIP and inflammation in pre-clinical and clinical studies. Pharmacological approaches targeting the GIP receptor (GIPR) with effects on inflammatory processes are discussed as well, including the latest GIP-based multi-target approaches. The impact of GIP on inflammation appears context-dependent and influenced by tissue-specific receptor expression and metabolic status. While GIP has been shown to exert both pro- and anti-inflammatory effects in experimental models, clinical data are still limited. The success of GIP/glucagon-like peptide-1 (GLP-1) dual agonists in improving glycometabolic and inflammatory outcomes, highlighted the need to disentangle the individual contributions of each pathway. GIPR remains a promising, yet understudied, target in immunometabolism. Future studies are needed to clarify the molecular mechanisms underpinning GIP’s immunomodulatory actions and evaluate the anti-inflammatory potential of GIP-targeting therapies in clinical settings.

## Introduction

Glucose-dependent insulinotropic polypeptide (GIP) is one of the main physiological incretin hormones [1]. Initially identified for the ability to inhibit gastric acid secretion, GIP was later characterized for its insulinotropic effects and its role in lipid storage and adipogenesis [2]. For a long time, it was considered metabolically redundant and largely overshadowed by its counterpart, glucagon-like peptide-1 (GLP-1), mainly due to reduced efficacy in individuals with type 2 diabetes mellitus (T2D) and obesity, and association with weight gain in some preclinical studies [3, 4]. The recent development and clinical success of dual GIP/GLP-1 receptor agonists have revitalized interest in GIP pharmacology. These agents have demonstrated remarkable metabolic benefits, including superior glycemic control and weight loss compared to GLP-1 receptor agonists alone in patients with T2D and obesity [5]. As a result, GIP’s physiological functions are being reexamined, with new evidence highlighting its roles in regulating also inflammatory processes. Recent preclinical studies have identified both pro- and anti-inflammatory actions of GIP, depending on disease context and experimental model [6–8]. GIP receptor (GIPR) has been found on various immune cells, although its functional significance remains unclear [9]. The potential for GIP to modulate inflammation is of particular relevance considering the growing recognition of chronic low-grade inflammation as a critical contributor to metabolic diseases, such as insulin resistance, T2D, obesity, atherosclerosis and non-alcoholic steatohepatitis [10]. Moreover, inflammatory pathways are also implicated in autoimmune forms of diabetes, as type 1 diabetes (T1D) and overlapping conditions like double diabetes (or type 1 diabetes with obesity), where immune dysregulation is coupled with metabolic dysfunction [11]. This review discusses the biological effects of GIP, evaluates the preclinical and clinical evidence linking GIP to both systemic and organ-specific inflammation, and explores the therapeutic implications of GIPR-targeting strategies in metabolic and inflammatory diseases. A focus on GIP role in inflammatory pathways within metabolically relevant tissues during metabolic diseases like T2D and obesity will be made in line with the growing interest of GIP targeting in these conditions.

### GIP biological effects

**Fig. 1:**
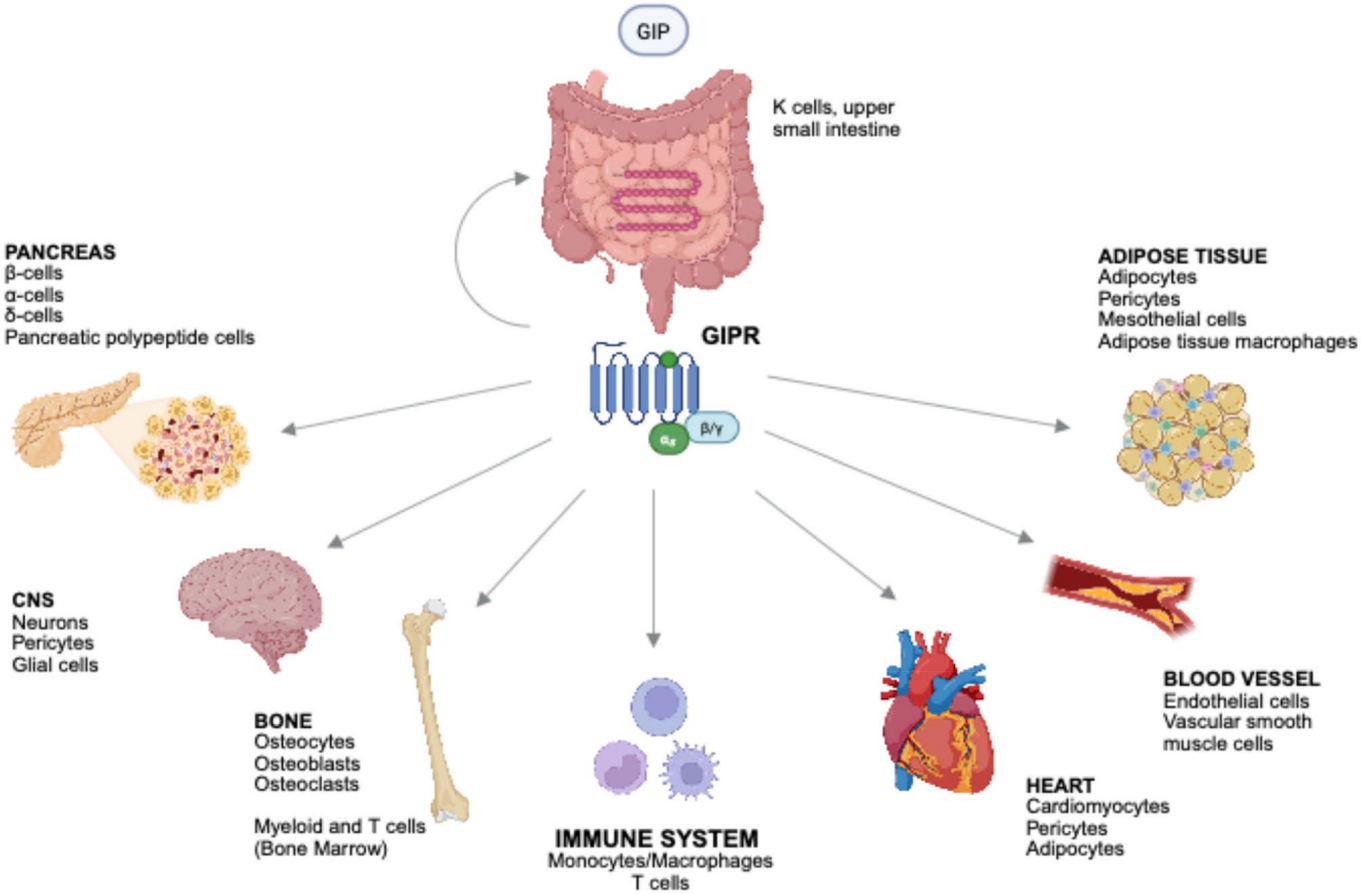
Expression of GIP receptor (GIPR) in human tissues

GIP is a 42-amino acid peptide hormone secreted by K cells in the proximal small intestine in response to fat and carbohydrate ingestion [12]. It acts by binding to a G protein-coupled receptor, which is expressed in multiple tissues accounting for its pleiotropic effects [2] (Fig. 1). GIP’s primary recognized function is to enhance β-cells glucose-dependent insulin secretion by increasing cyclic adenosine monophosphate levels [13]. This leads to the activation of protein kinase A and exchange protein directly activated by cAMP-2, which together potentiate insulin granule exocytosis [14]. In addition, GIP can promote β-cell survival by activating anti-apoptotic signaling cascades and support β-cell growth and proliferation [15, 16]. Importantly, GIPR expression in pancreatic islets appears to be dynamic and modulated by age, metabolic state, and glycemic control. GIPR downregulation was observed in chronic hyperglycemia potentially contributing to incretin resistance in subjects with T2D [17]. In contrast to GLP-1, GIP is known to stimulate glucagon release from pancreatic α-cells, particularly under hypoglycemic or euglycemic conditions [18]. Nevertheless, in insulin-deficient states as advanced T2D, the glucagonotropic action of GIP may predominate, exacerbating hyperglycemia and reducing its therapeutic efficacy [19]. In addition to its actions in the endocrine pancreas, GIP plays a pivotal role in lipid homeostasis. In mouse brown adipose tissue, GIP regulates thermogenesis-related genes and upregulates lipid, amino acid and glucose catabolic processes [20]. In the white adipose tissue, GIP promotes lipid uptake and lipogenesis through upregulation of fatty acid transporters and activation of lipoprotein lipase [21]. However, GIP lipogenic effects may become maladaptive under chronic energy excess and contribute to insulin resistance and weight gain. Indeed, chronic GIP stimulation has been associated with increased deposition of fat mass in animal models, whereas GIPR-deficient mice are resistant to diet-induced obesity [22]. In the cardiovascular system, GIP exerts complex and multifaceted effects mediated by the GIPR, which is expressed on endothelial cells, vascular smooth muscle cells and cardiomyocytes [23]. In preclinical models, acute GIP administration improves endothelial function via stimulation of endothelial nitric oxide synthase and enhanced bioavailability of nitric oxide, promoting vasodilation and mean arterial blood reduction [24]. In addition, GIP has been shown to attenuate atherogenesis [25]. Accordingly, lower GIP levels have been associated with poor cardiovascular outcomes in high-risk patients with acute myocardial infarction [26]. Among its multiple functions, GIP also contributes to post-prandial bone formation and inhibition of bone resorption, and it can act as a neuroactive hormone within the central nervous system [27]. Indeed, GIPR has been detected in key brain regions involved in energy homeostasis, reward processing and cognition, like the hypothalamus, hippocampus and brainstem, with some overlap with GLP-1R expression [28]. Central administration of GIP or long-acting GIP analogs in diet-induced obesity mice reduce their food intake and body weight. These effects are abolished in GIPR^−/−^ mice [29]. Moreover, GLP-1R and GIPR co-agonism has shown superior efficacy in reducing body weight compared with GLP-1 agonism alone, with effects being mediated through GIP receptor signaling in mice central nervous system [30]. In addition, GIP has demonstrated neuroprotective effects in preclinical models of neurodegenerative diseases, which display features of increased neuroinflammation also observed in T2D and insulin resistant patients [31]. Ultimately, GIP signaling appears to modulate brainstem circuits involved in emesis. In preclinical models, co-administration of GIP with GLP-1 receptor agonists reduced nausea-like behaviors and emesis. These findings may underlie the improved tolerability of dual GIP/GLP-1 agonists in humans [32]. Altogether, GIP exerts widespread biological activity across numerous organ systems, reflecting its multiple effects beyond glucose homeostasis.

### GIP and inflammation

Emerging evidence suggests that GIP may exert direct and indirect effects on inflammation [33]. These novel findings reveal an unexpected role for GIP in modulating inflammatory pathways, opening new perspectives on its physiological relevance and therapeutic potential.

#### Animal studies

Recent preclinical studies have revealed both pro- and anti-inflammatory actions of GIP, depending on tissue, disease context, and experimental model [34]. In monocytes and macrophages, GIPR activation decreases the production of pro-inflammatory cytokines such as TNF-α, IL-1β, IL-8 and IL-18 [35, 36]. Accordingly, increased bioavailability of GIP and GLP-1 by the dipeptidyl peptidase 4 inhibitor, sitagliptin, promotes anti-inflammatory polarization of Mϕ macrophages towards M2 macrophages and reduces mitochondrial reactive oxygen species production [37–39]. Moreover, lipopolysaccharides-stimulated RAW264.7 cells treated with the dipeptidyl peptidase 4 inhibitor, vildagliptin, or with the competitive incretin receptor binding inhibitor, mannose-6-phosphate, displayed reduction in TLR2 and TLR4 expression as well as in pro-inflammatory cytokines production [40]. GIP can regulate inflammation in obesity via modulation of myelopoiesis in bone marrow and expression of the pro-inflammatory S100 calcium-binding protein heterodimer S100A8/A9 in bone marrow and adipose tissue macrophages [8, 9]. Reduced adipose tissue infiltration of inflammatory Ly6C(hi) monocytes, F4/80(hi)CD11c^+^ macrophages, and IFN-γ-producing CD8^+^ and CD4^+^ T cells was shown after administration of the long-acting GIP analog [d-Ala(2)]GIP in diet-induced obesity mice. In addition, reduction in key inflammatory cytokines and chemokines, and increase in adiponectin release by adipocytes was observed [9]. Conversely, GIP stimulation increased monocyte chemoattractant protein 1 transcripts (MCP-1) in co-cultures of adipocytes and macrophages, indicating enhanced macrophages recruitment in the adipose tissue during obesity [35]. Similarly, intraperitoneal administration of GIP in obese db/db mice has been associated with increased monocyte chemoattractant protein 1, plasminogen activator inhibitor 1 and IL-6 production by the adipose tissue, at least in part mediated by upregulation of hypoxia-inducible factor-1α [6]. GIP signaling has been shown to modulate also vascular, brain and gut inflammation. GIP treatment attenuated atherosclerotic plaque inflammation in atherosclerosis-prone Apolipoprotein E-null (ApoE^−/−^) mice and stabilized the atherosclerotic plaque in diabetic mice [41–43]. On the other hand, infusion of GIP induced the expression of the proatherogenic cytokine osteopontin in mouse arteries via local release of endothelin 1 and activation of cAMP-response element binding protein [44]. GIP administration increased proinflammatory-related factors, such as IL-6 and suppressor of cytokine signaling 3, in the hypothalamus of high-fat diet-fed C57BL/6J male mice [45]. Conversely, GIP treatment has been demonstrated to alleviate 5-fluorouracil-induced gut inflammation [7]. The blockade of GIP signaling or GIPR deficiency reduced the accumulation of T regulatory cells in the adipose tissue and significantly reduced proinflammatory-related factors in the hypothalamus of high-fat diet-fed C57BL/6J male mice [45, 46]. In contrast, aortic atherosclerosis and mRNA transcripts of pro-inflammatory genes were increased in ApoE^−/−^:GIPR^−/−^ mice, and genetic deletion of GIPR exacerbated the proinflammatory response to 5-fluorouracil in murine small bowel [7, 47]. Finally, GIPR^−/−^ mice exhibited reduction of hematopoietic stem cells and CD45^+^ cells in the bone marrow, and of neutrophils, Ly6Chi/Lo monocytes, T cells and natural killer cells both in bone marrow and in circulation [48]. GIP can therefore be considered a significant modulator of several key inflammatory pathways occurring in both native and adaptive immune cells (i.e., monocytes/macrophages, neutrophils, NKT cells, microglia, myeloid and T cells) and non-immune cells (i.e., adipocytes, endothelial cells, neurons and stromal cells), although with some conflicting results that warrant further investigation (Tables 1 and 2).

**Table 1:** Pro-inflammatory effects of GIP

Cell type/tissue/organ	Model	Treatment	Effects	References
**Animal studies**				
Bone marrow myeloid cells	GIPR^-/-^ mice	GIPR KO in HFD	↑ myelopoiesis and S100A8/9	[8, 9]
Adipose tissue macrophages	GIPR^-/-^ mice	GIPR KO in HFD	↑ S100A8/9	[8, 9]
Macrophages	RAW264.7 cells	GIP treatment in co-culture with 3T3L1-adipocytes	↑ MCP-1	[35]
White adipocytes	3T3-L1 cells	GIP treatment	↑ MCP-1, PAI-1, IL-6 mediated by HIF-1α	[6]
White adipocytes	Obese db/db mice	GIP treatment	↑ MCP-1, PAI-1, IL-6 mediated by HIF-1α	[6]
Brown adipocytes	GIPR^-/-^ BAT mice	GIP antagonism	↓ PPAR-γ ◊ ↓ Tregs accumulation in adipose tissue	[46]
Arteries	NMRI; FVBN NFAT-luc; NFATc3^−/−^; Akita^+/−^ LDLr^−/−^ mice	GIP treatment	CREB activation and increase in ET-1 ◊ ↑ OPN	[44]
Hypothalamus	HFD-fed male mice	GIP treatment	↑ IL-6, SOCS-3	[45]
Small bowel	GIPR^-/-^ mice	GIP KO + 5FU treatment	↑ IL-1β, IL-10, IL-6, and TNF-α, CXCL-1, IFN-γ, S100A8	[7]
**Human studies**				
Macrophages	Primary human macrophages	GIP treatment in co-culture with primary human adipocytes	↑ MCP-1	[35]
White adipocytes	Human adipose tissue	GIP infusion	↑ MCP-1, MCP-2, IL-6	[35]
White adipocytes	Human subcutaneous preadipocytes-derived adipocytes	GIP treatment	↑ IL-6, IL-1β, IL-1Ra	[50]
Systemic effect	Obese humans	GIP infusion	↑ MCP-1	[35]
Systemic effect	Healthy humans	GIP treatment	CREB activation and increase in ET-1 ◊ ↑ OPN	[44]

*Abbreviations: *GIPR: glucose-dependent insulinotropic polypeptide receptor, KO: knockout, HFD: high-fat diet, GIP: glucose-dependent insulinotropic polypeptide, MCP: monocyte chemoattractant protein, HIF-1α:hypoxia-inducible factor-1α, PAI-1:plasminogen activator inhibitor 1, IL: interleukin, BAT: brown adipose tissue, PPAR: peroxisome proliferator-activated receptor, Tregs: T regulatory cells, TNF: tumor necrosis factor, NOS: nitric oxide synthase, NFAT-luc: nuclear factor of activated T-cells luciferase, LDLr: low-density lipoprotein receptor, CREB: cAMP-response element binding protein, ET-1:endothelin 1, OPN: osteopontin, SOCS: suppressor of cytokine signaling, 5FU:5-fluorouracil, CXCL: C-X-C motif chemokine ligand, IFN: interferon, IL-1Ra: IL-1 receptor antagonist

**Table 2:** Anti-inflammatory effects of GIP

Cell type/tissue/organ	Model	Treatment	Effects	References
**Animal studies**				
Hematopoietic stem cells; myeloid precursors; CD45^+^ cells in BM	GIPR^-/-^ mice	GIPR KO	↓ SDF-1, TLR, Notch signaling, cell number	[48]
Neutrophils; Ly6Chi/Lo monocytes; T cells; NKT cells	GIPR^-/-^ mice	GIPR KO	↓ cell number in BM and circulation	[48]
Macrophages	LPS-stimulated RAW264.7 cells	DPP4i (vildagliptin)	↑ cAMP/PKA ◊ suppression of NF-kB and MAPK ◊ ↓ IL-1β, TNF-α, IL-18, IL-8	[35]
Macrophages	Bone marrow-derived MØ from L929 cells	DPP4i (sitagliptin)	M1 ◊ M2 polarization and ↓ ROS	[37]
Macrophages	LPS-stimulated RAW264.7 cells	DPP4i (vildagliptin) or M6P (competitive incretin receptor binding inhibitor)	↓ TLR2, TLR4, pro-inflammatory cytokines	[40]
Macrophages	GIPR^+/+^ mice	GIP analog [D-Ala2]GIP vs. vehicle	↓ CD36 and ACAT ◊ ↓ foam cells formation	[42]
Adipose tissue	DIO mice	GIP analog [d-Ala(2)GIP]	↓ infiltration of inflammatory Ly6C(hi) monocytes, F4/80(hi)CD11^+^ macrophages, IFN-γ-producing CD8^+^ and CD4^+^ T cells, IFN-γ, IL-1β, TNF-α, CCL2, CCL8, CCL5; ↑ in adiponectin release	[9]
Arteries	ApoE^-/-^ mice	GIP treatment	↓ inflammation and macrophages activation	[41]
Arteries	ApoE^-/-^ mice or db/db mice	GIP treatment	↓ foam cells formation; maintenance of VSMCs contracted phenotype; ↑ collagen and thickness of fibrous plaque cap	[41]
Arteries	ApoE^-/-^:GIPR^-/-^ mice	ApoE and GIPR KO	↓ mRNA transcripts of inflammatory genes	[47]
Hypothalamus	HFD-fed male mice GIPR^+/+^	GIPR antibody	↓ IL-6, SOCS-3	[45]
Hypothalamus	HFD-fed male mice GIPR^-/-^	GIP KO	↓ IL-6, SOCS-3	[45]
Microglia	BV-2 cells and primary microglia	GIP treatment	Activation of PI3K/PKA◊ ↓ apoptosis and ROS; ↑ BDNF, GDNF, NGF, GPx-1 and SOD-1	[53]
Gut stromal CD146+ cells	C57BL/6J mice	GIP treatment	↓ 5FU-induced gut inflammation	[7]
Systemic effects	Immune cells-restricted GIPR^-/-^:S100A8/9^-/-^ mice	Immune cells-restricted GIPR and S100A8/9 KO in HFD	↓ systemic inflammation	[8, 9]
Systemic effect	Chow or HFD-fed mice	GIP analog [d-Ala(2)GIP]	↓ circulating neutrophils and pro-inflammatory Ly6C(hi) monocytes	[9]
**Human studies**				
Macrophages	Human subcutaneous fat biopsies of slightly obese patients	GIP treatment	↑ cAMP/PKA ◊ suppression of NF-kB and MAPK ◊ ↓ IL-1β, TNF-α, IL-18, IL-8	[35, 36]
Macrophages	U937 cells	GIP analog [D-Ala2]GIP vs. vehicle	↓ Cdk5-CD36 ◊ ↓ foam cells formation	[51]
Endothelial cells	HUVEC; ECV304; HPAEC; HAEC cells	GIP treatment	↑ AMPK and eNOS ◊ ↑ NO and cAMP ◊ ↓ ROS, AGEs, VCAM-1, ICAM-1, PAI-1	[52]

*Abbreviations: *BM: bone marrow, GIPR: glucose-dependent insulinotropic polypeptide receptor, KO: knockout, SDF-1:stromal cell-derived factor 1, TLR: toll-like receptor, NKT: natural killer T cells, LPS: lipopolysaccharides, cAMP: cyclic adenosine monophosphate, PKA: protein kinase A, NF-κB: nuclear factor kappa-light-chain-enhancer of activated B cells, MAPK: mitogen-activated protein kinase, IL: interleukin, TNF: tumor necrosis factor, MØ:naïve macrophage, M1:macrophage type 1, M2:macrophage type 2,, ROS: reactive oxygen species, DPP4i: dipeptidyl peptidase 4 inhibitors, M6P: mannose-6-phosphate, ACAT: A:cholesterol acyltransferase, DIO: diet-induced obesity, IFN: interferon, GIP: glucose-dependent insulinotropic polypeptide, ApoE: apolipoprotein E, VSMC: vascular smooth muscle cells, HFD: high-fat diet, SOCS: suppressor of cytokine signaling, PI3K: phosphoinositide 3-kinase, BDNF: brain-derived neurotrophic factor, GDNF: glial cell line-derived neurotrophic factor, NGF: nerve growth factor, GPx1:glutathione peroxidase 1, SOD-1:superoxide dismutase 1, 5FU:5-fluorouracil, HUVEC: human umbilical vein endothelial cells, HPAEC: human pulmonary artery endothelial cells, HAEC: human aortic endothelial cells, AMPK: adenosine monophosphate-activated protein kinase, eNOS: endothelial nitric oxide synthase, NO: nitric oxide, AGEs: advanced glycation end-products, VCAM-1:vascular cell adhesion molecule 1, ICAM-1:intercellular adhesion molecule 1, PAI-1:plasminogen activator inhibitor 1

#### Human studies

GIP receptor has been detected on various immune cells, however the distribution within the human immune system remains incompletely characterized as well as the functional significance [49]. MCP-1 transcripts were increased in co-cultures of human adipocytes and macrophages following GIP stimulation [35]. GIP treatment of human subcutaneous preadipocyte-derived adipocytes upregulated mRNA expression of IL-6, IL-1β, and the IL-1 receptor antagonist [50]. Moreover, GIP infusion in slightly obese human subjects has been associated with increased monocyte chemoattractant protein-1 and − 2, and IL-6 production by the adipose tissue [35]. Elevated concentrations of GIP were found in patients with atherosclerotic cardiovascular disease [41]. Indeed, infusion of GIP increases the plasmatic concentrations of osteopontin in healthy individuals [44]. By contrast, human U937 macrophages treated with [D-Ala2]GIP(1–42) showed significantly lower foam cell formation and CD36 gene expression compared to untreated controls [51]. GIP can also enhance nitric oxide production and reduce reactive oxygen species generation, advanced glycation end-products signaling, and vascular cell adhesion molecule 1, intercellular adhesion molecule 1, and plasminogen activator inhibitor 1 levels in endothelial cells [52]. Indeed, in the post-prandial phase, incretin hormones promote vasodilation, supporting tissue perfusion and increased metabolic demands. This effect is reduced in people with obesity. Particularly, reduced blood flow following decreased GIP effects during obesity leads to adipose tissue inflammation, promoting further metabolic and cardiovascular disfunction [30]. Ultimately, GIPR is expressed by primary human microglia and astrocytes, potentially playing several homeostatic functions in the immune cells of the brain [53]. Collectively, GIP orchestrates complex and multifaceted systemic and local immunomodulatory networks in a context-dependent manner (Tables 1 and 2). These immunomodulatory actions suggest a potential role for GIP in metabolic inflammation and autoimmune diseases. In fact, previous studies have reported a link between GIP and several autoimmune disorders, with GIP peptide showing positive effects on bone formation and energy homeostasis in rheumatoid arthritis and reduced expression in systemic lupus erythematosus and inflammatory bowel disease [34].

### GIP targeting

Given the functional duality of GIP peptide in modulating several inflammatory pathways, targeting GIP either through agonist or antagonist compounds, can impact both physiological and pathological inflammatory responses. Data on GIP stand-alone agonism and antagonism derive mainly from preclinical studies and showed conflicting metabolic and inflammatory outcomes according to the disease model (Tables 1 and 2), which has so far hindered their translation into clinical practice. In contrast, multiple-target therapies, like GIP/GLP-1 dual agonists, are showing promising results also in human studies, representing a novel frontier in the treatment of various diseases [54]. As shown for improvements in glycemic control and body weight reduction, the combination of GIP targeting with the well-established anti-inflammatory properties of GLP-1 receptor agonists (GLP-1RAs) may potentiate the overall immunomodulatory action of these drugs [55–60]. Indeed, solid evidence exists regarding the anti-inflammatory properties of GLP-1R agonist in autoimmune and inflammatory conditions such as type 1 diabetes, multiple sclerosis, rheumatoid arthritis, psoriasis, systemic lupus erythematosus, inflammatory bowel disease and cancer [46]. In contrast, less is known about GIP targeting in such diseases, although with encouraging, yet still incomplete, data coming from GIP/GLP-1 receptors agonism. Tirzepatide has been shown to be safe and effective in autoimmune diabetes [61], and is currently under investigation following (NCT06857942 and NCT06864026) or in combination with the anti-IL-17 A ixekizumab (NCT06588296 and NCT06588283), or the anti-IL-23 mirikizumab (NCT06937099) in obese or overweight patients with psoriatic arthritis, plaque psoriasis or active Crohn’s disease, respectively. Evidence from these studies, along with data potentially arising from the use of tirzepatide in patients with T2D, obesity and coexisting autoimmune diseases, could help fill the current knowledge gap regarding GIP targeting in chronic inflammatory conditions.

#### Animal studies

The dual GIP/GLP-1 receptor agonist tirzepatide has shown several anti-inflammatory properties (Table 3). Intraperitoneal injection of tirzepatide in mice with streptozotocin-induced diabetic nephropathy showed reduction in advanced glycation end-products and pro-inflammatory cytokines in both serum and kidney homogenates [62]. Tirzepatide treatment significantly mitigated the infiltration of pro-inflammatory M1 adipose tissue macrophages within the adipose tissue and reduced the levels of inflammatory cytokines in high-fat diet-fed mice [63]. Treatment with tirzepatide demonstrated the ability to reverse intestinal dysbiosis, repair intestinal barrier integrity and reduce gut inflammation in diet-induced obesity diabetic mice [64]. Moreover, tirzepatide exhibited a strong beneficial effect on hepatic fat deposition and inflammation in the liver of diabetic db/db mice and high-fat diet-fed rats [65]. Tirzepatide attenuated also lipopolysaccharides- and doxorubicin-induced cardiac dysfunction in mice and H9c2 cells by reducing oxidative stress and cardiac protein levels of TNF-α, IL-6, and IL-1β [66, 67]. Finally, tirzepatide exerted neuroprotection and anti-inflammatory effects in the hippocampus of diabetic rats, as well as in key brain areas of Alzheimer and Parkinson’s disease animal models [68–70]. The GLP-1/GIP dual-receptor agonists DA5-CH and DA-JC1 can inhibit the NF-κB inflammatory pathway in mouse and rat models of Parkinson’s disease more effectively than GLP-1 single-receptor agonist [71–73]. DA4-JC has shown protective and anti-inflammatory properties in mouse and rat models of Alzheimer’s disease [74]. DA3-CH could mitigate pilocarpine-induced neuro-inflammation, mitochondrial apoptosis and neuronal loss in a rat model of epileptogenesis [75, 76]. In addition, the novel dual GLP-1/GIP receptor agonist AP5 effectively reduced hyperglycemia, reactive oxygen species production, oxidative stress and inflammatory markers in a rodent model of diabetic cardiomyopathy [77]. The latest frontier in GIP targeting is constituted by triple or quadruple agents acting on GIPR and GLP-1R, together with other metabolically relevant targets like glucagon, amylin or calcitonin. The triple GLP-1, GIP and glucagon receptor agonist, retatrutide (LY3437943), demonstrated higher reduction of pro-inflammatory cytokines (TNF-α, caspase-1, and NLRP3) and pro-fibrotic factors (fibronectin, α-SMA, and collagen I) in kidneys of db/db mice, and better effects on the intestinal microbiota compared to liraglutide and tirzepatide [78]. GIP targeting, particularly if combined with GLP-1 receptor agonism, emerges therefore as a promising strategy to mitigate inflammation in several chronic inflammatory conditions.

**Table 3:** Anti-inflammatory effects of the GIPR/GLP-1R dual agonist Tirzepatide (TRZ)

Intervention	Target/mechanism of action	Preclinical/clinical model	Anti-inflammatory effects	References
**Animal studies**				
Tirzepatide	GIPR agonism + GLP-1R agonism	STZ-induced diabetic nephropathy mice	↓ AGEs, TNF-α, IL-1β, and IL-6 (serum and kidney)	[62]
Tirzepatide vs. carrier	GIPR agonism + GLP-1R agonism	HFD-fed mice	↓ infiltration of M1 ATMs in adipose tissue, ↓ TNF-α, IL-6, MCP-1, IFN-γ	[63]
Tirzepatide vs. vehicle	GIPR agonism + GLP-1R agonism	DIO diabetic ovariectomized mice	Reverse intestinal dysbiosis; repair intestinal barrier integrity; ↓ macrophage activation and gut inflammation	[64]
TRZ vs. semaglutide vs. PBS	GIPR agonism + GLP-1R agonism (TRZ); GLP-1R agonism (semaglutide)	Diabetic db/db mice	↓ hepatic fat deposition, MCP1, chemokines-related genes vs. semaglutide and placebo; ↓ liver M1/M2 ratio vs. placebo	[65]
Tirzepatide vs. liraglutide vs. vehicle	GIPR agonism + GLP-1R agonism (TRZ); GLP-1R agonism (liraglutide)	Angiotensin II-induced heart failure mice model	↓ cardiac fibrosis (TRZ and liraglutide); ↓ systemic CRP (TRZ)	[66]
Tirzepatide vs. saline	GIPR agonism + GLP-1R agonism	Doxorubicin-induced cardiac injury mice model	↓ ROS, 4-HNE, IL-1β, IL-6, TNF-α; ↑ SOD and CAT activity	[67]
Tirzepatide vs. citrate buffer	GIPR agonism + GLP-1R agonism	HFD and STZ-induced diabetic rats	↓ amyloid beta (Aβ) deposition, TNF-α, IL-6, IL-1β in the hippocampus	[68]
Tirzepatide vs. exendin-4 vs. PBS	GIPR agonism + GLP-1R agonism (TRZ); GLP-1R agonism (exendin-4)	Rotenone-induced toxicity model in rats (PD model)	↓ TNF-α, IL-6, oxidative stress and alpha-synuclein aggregation vs. PBS	[69]
Tirzepatide vs. saline	GIPR agonism + GLP-1R agonism	APP/PS1 mice (AD model)	↓ amyloid beta (Aβ) deposition and ROS	[70]
**Human studies**				
Tirzepatide vs. dulaglutide vs. placebo	GIPR agonism + GLP-1R agonism (TRZ); GLP-1R agonism (dulaglutide)	Phase IIb clinical trial in T2D patients	↓ YKL-40, leptin, ICAM‐1, GDF-15 vs. baseline; ↓ YKL‐40 and leptin vs. placebo and dulaglutide; ↓ ICAM‐1 vs. placebo and dulaglutide; ↓ hsCRP vs. baseline and placebo	[79]
Tirzepatide vs. placebo	GIPR agonism + GLP-1R agonism	Phase III clinical trial in patients with HFpEF and obesity (SUMMIT trial)	↓ hsCRP	[80]
Tirzepatide or GIP	GIPR agonism + GLP-1R agonism (TRZ); GIPR agonism (GIP)	Mature human pancreatic adipose tissue organoids	↓ MCP-1, adiponectin, IL-6 (TRZ); ↓ IL-1β (TRZ and GIP)	[81]
Tirzepatide or dulaglutide or semaglutide	GIPR agonism + GLP-1R agonism (TRZ); GLP-1R agonism (dulaglutide; semaglutide)	Retrospective cohort of T2D patients with MASLD	↓ liver fat, fibrosis and hsCRP levels	[82]
Tirzepatide or [D-Ala2]-GIP with or without liraglutide	GIPR agonism + GLP-1R agonism (TRZ); GIPR agonism [D-Ala2]-GIP; GLP-1R agonism (liraglutide)	Human islet microtissues exposed to proinflammatory cytokines to mimic T1D	Restore cytokine-induced alpha cell impairment	[83]

*Abbreviations: *GIPR: glucose-dependent insulinotropic polypeptide receptor, GLP-1R: glucagon-like peptide-1 receptor, HFpEF: heart failure with preserved ejection fraction, hsCRP: high-sensitivity C‐reactive protein, STZ: streptozotocin, AGEs: advanced glycation end-products, TNF: tumor necrosis factor, IL: interleukin, HFD: high fat diet, M1:macrophage type 1, M2:macrophage type 2, ATMs: adipose tissue macrophages, MCP-1:monocyte chemoattractant protein-1, IFN: interferon, DIO: diet-induced obesity, CRP: C‐reactive protein, ROS: reactive oxygen species, 4-HNE:4-Hydroxynonenal, SOD: superoxide dismutase, CAT: catalase, PBS: phosphate-buffered saline, PD: Parkinson’s disease, AD: Alzheimer’s disease, YKL‐40:chitinase-3-like protein 1, ICAM‐1:intercellular adhesion molecule 1, GDF-15:growth differentiation factor 15, GIP: glucose-dependent insulinotropic polypeptide, MASLD: metabolic dysfunction-associated steatotic liver disease

#### Human studies

Anti-inflammatory properties of tirzepatide have been shown also in human studies (Table 3). A post-hoc analysis of a phase 2b clinical trial assessing efficacy of tirzepatide versus placebo or dulagutide in patients with T2D, demonstrated that tirzepatide could decrease YKL-40, leptin and intercellular adhesion molecule 1 levels versus baseline, placebo and dulaglutide, and high‐sensitivity C‐reactive protein versus baseline and placebo [79]. High‐sensitivity C‐reactive protein was found to be decreased by tirzepatide also in a post-hoc analysis of the SUMMIT trial in patients with heart failure and obesity [80]. Functional mature human pancreatic adipose tissue organoids exposed to tirzepatide for 24 h exhibit reduction in monocyte chemoattractant protein 1 expression and in adiponectin, IL-1β and IL-6 release [81]. Tirzepatide demonstrated positive effects on hepatic fat deposition and liver inflammation also in T2D patients with metabolic dysfunction-associated steatotic liver disease [82]. Additionally, tirzepatide has been shown to restore cytokine-induced alpha cell impairment in a model of T1D [83]. Surprisingly, also the GIPR antagonism showed anti-inflammatory ability. The long-acting peptide-antibody conjugate combining GLP-1 receptor agonism with GIP receptor antagonism, maridebart cafraglutide (MariTide or AMG133), has demonstrated to decrease high‐sensitivity C‐reactive protein levels together with body weight and glycated hemoglobin compared to placebo in patients with obesity alone or with obesity and T2D [84]. Some hypotheses have been proposed to explain the paradoxical observation that both GIPR agonism and antagonism can lead to similar outcomes (i.e., body weight reduction, improved glycemic control and reduced inflammation). Chronic GIPR agonism may lead to receptor desensitization, functionally mimicking antagonism. Conversely, GIPR antagonism may indirectly enhance GLP-1R activity, possibly through compensatory mechanisms [85]. At the same time, the imbalanced agonism towards GIPR exerted by tirzepatide together with the biased agonism towards GLP1-R, which favors cAMP generation over β-arrestin recruitment, could reduce GLP-1R desensitization and potentiate its activity [86]. Moreover, evidences demonstrated that GLP1-R and GIPR can form heteromers, and their signaling can interact, so that GIP-1R may potentiate GIP signaling and GIPR may sensitize GLP-1R signaling [87]. Therefore, tirzepatide exerts a synergistic effect that goes beyond the additive activity of GIPR and GLP-1R. This could potentially extend to its anti-inflammatory actions, although dedicated studies are currently lacking in the literature. Finally, tirzepatide, retatrutide and the triple agonist HM15211 (efocipegtrutide) have shown a strong anti-inflammatory and anti-fibrotic effect in the liver during metabolic dysfunction associated steatotic liver disease and metabolic dysfunction-associated steatohepatitis [88]. Altogether, these findings highlight the promising therapeutic potential of GIP-based multi-target approaches in modulating systemic inflammation alongside metabolic benefits (Tables 3 and 4). Some limitations should be acknowledged, which restrict the generalizability of the current evidence and highlight the need for further research on GIPR targeting. Firstly, most findings derive from animal or cell culture models, constrained by species-specific differences and uncertain translatability to humans. For example, weight loss induced by tirzepatide seems to result from partially different mechanisms in mice and humans, with suppression of food intake and attenuated metabolic adaptation in obese mice vs. decreased caloric intake without preservation of previous energy expenditure levels in humans [89]. Secondly, existing clinical studies are few, typically involving small sample sizes, short duration, and restricted populations, with large randomized controlled trials still lacking. Moreover, assessed outcomes vary widely, ranging from cytokines to gene expression and clinical parameters, with poor standardization across studies. Interpretation is further complicated by the context-dependent actions of GIP, which may exert pro- or anti-inflammatory effects depending on tissue type, metabolic status, and comorbidities, often producing contradictory results. Finally, the influence of concomitant hormones, cytokines, and environmental factors limits the possibility to isolate GIP-specific effects.

**Table 4:** Anti-inflammatory effects of novel GIP-target therapies

Intervention	Target/mechanism of action	Preclinical/clinical model	Anti-inflammatory effects	References
**Novel GIPR/GLP-1R dual agonists**				
DA5-CH vs. NLY01	GIPR agonism + GLP-1R agonism (DA5-CH); GLP-1R agonism (NLY01)	MPTP-PD mouse model	↓ TLR4, Iba-1, GFAP, NF-κB, TNF-α, TGF-β1, IL-6, IL-Iβ	[71]
DA-CH5 vs. exendin-4 or liraglutide	GIPR agonism + GLP-1R agonism (DA-CH5); GLP-1R agonism (exendin-4)	6-OHDA-unilaterally lesioned PD rat model; A53T tg mouse model of PD	↓ α-synuclein, TNF-α, IL-1β, apoptotic processes; protection of mitochondrial functions	[72]
DA-CH5 vs. exendin-4 or liraglutide	GIPR agonism + GLP-1R agonism (DA5-CH); GLP-1R agonism (exendin-4 and liraglutide)	MPTP-PD mouse model	↓ microglia and astrocyte activation; ↑ mitochondrial activity; normalization of autophagy vs. liraglutide	[73]
DA4-JC vs. liraglutide	GIPR agonism + GLP-1R agonism (DA-CH5); GLP-1R agonism (liraglutide)	APP/PS1 mice (AD model)	↓ amyloid plaques, TNF-α, IL-1β in the brain	[74]
DA3-CH vs. saline	GIPR agonism + GLP-1R agonism	Pilocarpine-induced rat model of epileptogenesis	↓ astrogliosis and microgliosis, TNF-α, IL-1β, ↓ apoptosis in the hippocampus	[75]
DA3-CH vs. liraglutide	GIPR agonism + GLP-1R agonism (DA3-CH); GLP-1R agonism (liraglutide)	MPTP-PD mouse model	↓ activated microglia and astrocytes	[76]
AP5 vs. GIP or GLP-1 vs. saline	GIPR agonism + GLP-1R agonism (AP5) or GIP or GLP-1 single agonism	Diabetic cardiomyopathy mice model	↓ ROS, TNF-α, IL-1β, NF-κB vs. single agonism and placebo	[77]
**GIPR antagonists/GLP1-R agonists**				
Maritide (AMG133) vs. placebo	GIPR antagonism + GLP-1R agonism	Phase II clinical trials in patients with obesity alone or with obesity and T2D	↓ hsCRP	[84]
**GIPR/GLP-1R/Glucagon receptor triple agonists**				
Retatrutide (LY3437943) vs. liraglutide vs. tirzepatide	GIPR antagonism + GLP-1R agonism + glucagon agonism (retatrutide); GLP-1R agonism (liraglutide); GIPR antagonism + GLP-1R agonism (TRZ)	db/db mice	↓ TNF-α, caspase-1, NLRP3, fibronectin, α-SMA, collagen I (kidneys); ↑ of serum butyrate (intestine) vs. liraglutide and TRZ	[78]
Retatrutride or HM15211 (efocipegtrutide) or TRZ	GIPR antagonism + GLP-1R agonism + glucagon agonism (retatrutide and HM15211); GIPR agonism + GLP-1R agonism (TRZ)	Phase II clinical trials in patients with NASH	↓ of liver inflammation	[88]

*Abbreviations*: GIPR: glucose-dependent insulinotropic polypeptide receptor, GLP-1R: glucagon-like peptide-1 receptor, hsCRP: high-sensitivity C‐reactive protein, TNF: tumor necrosis factor, IL: interleukin, ROS: reactive oxygen species, PD: Parkinson’s disease, AD: Alzheimer’s disease GIP: glucose-dependent insulinotropic polypeptide, MPTP: 1-methyl-4-phenyl-1,2,3,6-tetrahydropyridine, TLR4: toll-like receptor 4, Iba-1: ionized calcium-binding adaptor molecule 1, GFAP: glial fibrillary acidic protein, NF-κB: nuclear factor kappa-light-chain-enhancer of activated B cells, TGF: transforming growth factor, 6-OHDA: 6-hydroxydopamine, GLP-1: glucagon-like peptide-1, NLRP3: NLR family pyrin domain containing 3, α-SMA: alpha smooth muscle actin, NASH: non-alcoholic steatohepatitis

## Conclusions

Glucose-dependent insulinotropic polypeptide emerges as a modulator of several inflammatory pathways and a promising target in metabolic and inflammatory diseases. Despite the complexity and sometimes conflicting preclinical data regarding selective GIP agonism or antagonism, dual- and multi-target therapies combining GIP and GLP-1 modulation have demonstrated significant clinical potential in reducing systemic inflammation. These findings open new avenues for applying these agents in broader clinical contexts, including autoimmune, neurodegenerative and chronic inflammatory diseases. More research is needed to deeply understand GIP properties in regulating inflammation especially in the clinical setting in order to unlock its full clinical potential.

## Data Availability

Data sharing is not applicable to this article as no new data were created or analyzed in this study.
